# A Pilot Study of Behavioral, Physiological, and Subjective Responses to Varying Mental Effort Requirements in Attention-Deficit/Hyperactivity Disorder

**DOI:** 10.3389/fpsyg.2018.02769

**Published:** 2019-01-11

**Authors:** Gabry W. Mies, Pieter Moors, Edmund J. Sonuga-Barke, Saskia van der Oord, Jan R. Wiersema, Anouk Scheres, Jurgen Lemiere, Marina Danckaerts

**Affiliations:** ^1^Center for Developmental Psychiatry, UPC-KU Leuven, Leuven, Belgium; ^2^Behavioural Science Institute, Radboud University, Nijmegen, Netherlands; ^3^Laboratory for Experimental Psychology, Department of Brain and Cognition, KU Leuven, Leuven, Belgium; ^4^Institute of Psychiatry, Psychology and Neuroscience, King’s College London, London, United Kingdom; ^5^Department of Experimental Clinical and Health Psychology, Ghent University, Ghent, Belgium; ^6^Department of Clinical Psychology, KU Leuven, Leuven, Belgium; ^7^Department of Developmental Psychology, University of Amsterdam, Amsterdam, Netherlands

**Keywords:** attention-deficit/hyperactivity disorder, mental effort, pupil dilation, working memory, *N*-back task, effort discounting

## Abstract

**Background:** Attention-deficit/hyperactivity disorder (ADHD) is presumed to involve mental effort application difficulties. To test this assumption, we manipulated task difficulty and measured behavioral, as well as subjective and psychophysiological indices of effort.

**Methods:** Fifteen adolescent ADHD boys and 16 controls performed two tasks. First, subjective estimates and behavioral and pupillary measures of effort were recorded across five levels of *N*-back task difficulties. Second, effort discounting was assessed. In the latter, participants made repeated choices between performing a difficult *N*-back task for a high reward versus an easier *N*-back task for a smaller reward.

**Results:** Increasing task difficulty led to similar deteriorations in performance for both groups – although ADHD participants performed more poorly at all difficulty levels than controls. While ADHD and control participants rated the tasks equally difficult and discounted effort similarly, those with ADHD displayed slightly different pupil dilation patterns with increasing task difficulty.

**Conclusion:** The behavioral results did not provide evidence for mental effort problems in adolescent boys with ADHD. The subtle physiological effects, however, suggest that adolescents with ADHD may allocate effort in a different way than controls.

## Introduction

Attention-deficit/hyperactivity disorder (ADHD) is a common life-span neuro-developmental disorder, characterized by inappropriate levels of inattention and/or hyperactivity/impulsivity. Difficulties with the application of mental effort are implicated in both the clinical formulation and neuropsychological explanations of ADHD, but has been underexamined empirically. At the clinical level, a core symptom of DSM-5 diagnosis is “Avoids, dislikes, or is reluctant to engage in tasks that require sustained mental effort (such as schoolwork or homework).” At a neuropsychological level, the cognitive-energetic model (e.g., Sergeant, 2005; Van der Meere, 2005; Sonuga-Barke et al., 2010) proposes that poor task performance in individuals with ADHD may stem from problems in applying effort required for regulating physiological state to meet fluctuating environmental demands. In dull, boring, and under-stimulating settings children with ADHD, compared to other children, have difficulty increasing their arousal and activation levels to maintain optimal task engagement. In highly stimulating and exciting environments, they have difficulty constraining their states of over-arousal and -activation (state regulation account; Sergeant, 2005; Van der Meere, 2005; Sonuga-Barke et al., 2010). These problems lead to failures to maintain an optimal energetic disposition (Sonuga-Barke et al., 2010). Alternatively, individuals with ADHD may be capable of allocating effort effectively, but may simply be less motivated to do so (e.g., Morsink et al., 2017). There is plenty of clinical anecdotal and some experimental evidence that individuals with ADHD have no problems applying effort when they find tasks interesting (e.g., Dovis et al., 2012; Strand et al., 2012). This chimes with recent theories of mental effort that take psychological factors such as motivation into account by proposing the importance of cost–benefit consideration during the process by which individuals assign resources when performing tasks (e.g., Kurzban et al., 2013), i.e., the cost of performance is weighed against the expected value of its outcome (Kurzban et al., 2013; Westbrook et al., 2013; Botvinick and Braver, 2015; Massar et al., 2016). In general, people are prone to avoid cognitive effort and will only invest the required effort when the prospective benefits outweigh the anticipated investment (Kool et al., 2010). This explains why extrinsic reward can boost performance through increased effort (Massar et al., 2016). It is possible that under some circumstances the subjective cost of effort is too high for individuals with ADHD leading them to decrease effort application.

What is the evidence implicating effort allocation problems in ADHD? In a general sense, children with ADHD perform more poorly than typically developing children on effort intensive tasks that require executive control or working memory (for meta-analyses see Martinussen et al., 2005; Willcutt et al., 2005), effects that become more marked as task demands increase (Vaurio et al., 2009; Spronk et al., 2013; but see Gomarus et al., 2009; Van De Voorde et al., 2011). More specific support for the state regulation account (or cognitive energetic accounts) comes from experiments in which event rate is manipulated (i.e., the speed at which stimuli are presented). In a meta-analysis, Metin et al. (2012) found that participants with ADHD performed worse than controls under both slow and fast event rates compared to medium event rates. More specifically, under slow event rates in particular, participants with ADHD showed slower reaction times than controls, while under fast event rates, they showed elevated rates of errors of commission. A study by the same group found evidence of an accentuated inverted-U relationship across four levels of event rate on reaction time variability in ADHD (Metin et al., 2016): ADHD was associated with greater variability at both very long and very short rates. This variation as a function of event rate has been explained in terms of a deficit in the application of effort in situations with a suboptimal energetic state (e.g., Wiersema et al., 2006a; Sonuga-Barke et al., 2010; Metin et al., 2012, 2016; Johnstone and Galletta, 2013).

Electrophysiological studies have attempted to tie the effects of performance decrements in ADHD (e.g., as seen in event rate studies) to underlying energetic factors. For instance, reduced parietal P3 amplitudes have been found in individuals with ADHD compared to controls in situations with a suboptimal energetic state (Wiersema et al., 2006a,b; but see Johnstone and Galletta, 2013), as well as in other effortful tasks (e.g., Jonkman et al., 2000; Kim et al., 2014; Samyn et al., 2014). Since the P3 is an event-related brain potential component that is thought to reflect neural activity related to attention processes (Polich and Kok, 1995) and has thus been suggested to reflect the amount of effort invested in a task (Carrillo-de-la-Pena and Cadaveira, 2000; Kok, 2001), these findings suggest less effort allocation in ADHD. In electrophysiological studies in healthy subjects, P3 amplitudes increase when performance is rewarded (Rosch and Hawk, 2013), in line with the idea that reinforcement improves task performance through improved effort allocation.

The current study attempts to directly examine effort application in ADHD across multiple levels of measurement. It had two parts. In the first part of the study, we examined the impact of varying the demands for effort on a working memory task on objective and subjective indicators of effort demand and application. Our physiological measure of effort application was pupil dilation. Pupil dilation is a marker of autonomic nervous system activity – an increase in pupil size correlates with more sympathetic activity and greater mental effort, and pupil constriction with increased parasympathetic activity and less effort (Steinhauer et al., 2004). The amount of resources someone mobilizes during a task is thus reflected in a change in pupil size, that is, pupil size has been found to increase as a function of task difficulty (e.g., Kahneman and Beatty, 1966; Karatekin et al., 2004; Piquado et al., 2010) and has been considered as one of the best effort indices (Mulder, 1986). Only a few studies so far have used pupil dilation to examine working memory impairments in ADHD. Karatekin et al. (2009) found that ADHD was associated with reduced pupil dilation during a spatial working memory task, as well as worse performance, suggesting reduced effort application. Wainstein et al. (2017) also found reduced pupil dilation and worse performance during a spatial working memory task in children with ADHD, specifically when they were off medication. Both studies, however, only included two levels of task difficulty. This design may not be sufficient to fully model task difficulty effects in ADHD. In addition, these studies did not record children’s subjective perception of the levels of mental effort demanded, or the amount they felt they applied. Interestingly, a recent study found that individuals at risk for ADHD reported higher mental effort and discomfort during a working memory task than those not at risk, even though both groups performed equally on the task (Hsu et al., 2017).

In the second part of the study, we tested whether individuals with ADHD discount the (monetary) value associated with performing more effortful tasks more steeply than controls, as the difficulty – and thus effort required – increases. It makes perfect sense that if individuals with ADHD either require more effort to perform tasks or have difficulty mobilizing effort to solve demanding tasks, that such tasks will acquire a negative motivational significance over time and that they will choose to avoid or escape effort where they can more readily than typically developing controls (Sonuga-Barke et al., 2010). Thus, an emerging pattern of effort aversion might be predicted for ADHD individuals [see Sonuga-Barke (2005); Marco et al. (2009); and Van Dessel et al. (2018) for parallels with delay aversion]. To test this, we used an effort-related analog of a delay discounting task (i.e., an effort discounting task) where participants had to make multiple choices between performing a difficult *N*-back task (of varying difficulty) associated with a large monetary reward, and performing an easy *N*-back task associated with a smaller reward (of varying amount) (see also Westbrook et al., 2013).

We made the following predictions: (1) that increasing the level of task difficulty would lead to a steeper decline in performance in individuals with ADHD than controls; (2) that controls would show the expected increase in pupil dilation as task difficulty increased, and that this pattern would be less marked in individuals with ADHD (consistent with the idea that they are having difficulty mobilizing effort with increasing task demands); (3) that with increasing task difficulty individuals with ADHD would experience increasingly higher mental demand than controls; and (4) that individuals with ADHD would tend to choose less effortful tasks more often than controls, even if that meant earning less money (i.e., they would discount high effort-related rewards more than controls).

## Materials and Methods

### Participants

Sixteen boys with ADHD and 17 controls (aged 12–17 years) took part. The boys with ADHD were recruited through the Child and Adolescent Psychiatry department of UPC KU Leuven, and typically developing controls were recruited through schools, sports, and youth organizations, and other participants (e.g., siblings and friends). All participants and their parents gave written informed consent prior to participation, and the study was approved by the local medical ethics committee (UZ Leuven). All boys in the ADHD group had a pre-existing clinical diagnosis of ADHD combined presentation, and met diagnostic criteria of combined type ADHD on the Schedule for Affective disorders and Schizophrenia for School-Age Children (K-SADS; Kaufman et al., 1997) at the time of participation in another study (Van Dessel et al., 2018) [one had comorbid Oppositional Defiant Disorder (ODD)].

All participants were required to have normal, or corrected to normal, vision. Exclusion criteria for both groups were neurological illness, use of psychotropic medication other than methylphenidate, use of beta-blocking agents, learning disorders (e.g., dyslexia), IQ < 80 estimated on the basis of four Wechsler Intelligence Scale for Children (WISC-III-NL; Kort et al., 2002) subtests (Block Design, Vocabulary, Similarities, and Picture Arrangements), and comorbid psychiatric disorders [except for ODD and Conduct Disorder (CD) in the ADHD group]. Participants with ADHD who took methylphenidate (*n* = 9) were asked to discontinue their medication at least 24 h before assessment. All participants were asked to refrain from coffee or other caffeine-containing substances at least 2 h before assessment.

### Questionnaires

Parents completed the Disruptive Behavior Disorder Rating Scale (DBDRS; Dutch translation by Oosterlaan et al., 2000) to assess current ADHD symptom severity of their child, and the Child Behavior Checklist (CBCL; Achenbach and Rescorla, 2001) to assess internalizing and externalizing problem behavior. Participants completed a self-developed health questionnaire double-checking exclusion criteria (e.g., neurological illness, brain trauma, and medication use) and assessing other potentially relevant health concerns, the Behavioral Inhibition/Approach System Scales (BIS/BAS; Carver and White, 1994; Van den Berg et al., 2010) assessing reward sensitivity, and the revised Need for Cognition Scale (NCS; Cacioppo et al., 1984) to assess the extent to which individuals enjoy and engage in cognitively demanding tasks.

### Part I: *N*-Back Task With Varying Levels of Task Difficulty

The *N*-back task was based on the task used by Westbrook et al. (2013). Letters (consonants) were subsequently shown on the screen (max 1.5 s), intertwined with fixation underscores (_) (1 s). Participants had to indicate with a button press whether each letter was the same as the letter presented *N*-back (target letter), or different (non-target letter). Five levels of *N*-back difficulty (1- to 5-back) were administered, presented in 50-s blocks. Each block consisted of 20 trials, including 5 target and 15 non-target letters, of which 4 were lures (i.e., items within *N*+2, but not exactly *N* positions before last presentation). Participants were required to respond while the letter was shown on the screen, i.e., within 1.5 s: “1” for target letters, “3” for non-target letters, using the numerical part of a keyboard. Inter-stimulus intervals were fixed at 2.5 s. Each first 50-s block was preceded by a 3 s baseline (fixation), and each level of difficulty was presented twice in a row with a 10 s break (fixation) in between. Participants were informed of the level of difficulty of each double block just before it started. They did not know in advance how many levels of difficulty there were. The first task (1-back) was preceded by a practice block to get acquainted with the task. No practice blocks were used for subsequent levels of difficulty. All levels of difficulty were presented four times (two double blocks), and a fixed order was used for all individuals. To prevent that fatigue effects due to time-on-task would interfere with level of difficulty, the order of administration of each level of difficulty was as follows: participants performed a double block of the 1-back task, followed by a double block of the 3-, 5-, 2-, and 4-back task (first run). After these 10 blocks, participants had a 10–15-min break, followed by another round (second run) with the fixed order of double blocks of 2-, 4-, 1-, 5-, and 3-back. Total time was approximately 40 min.

### Pupil Measurements

During the *N*-back task pupil size was continuously measured by means of an eye-tracker (SMI RED-m, SensoMotoric Instruments, Berlin). Participants were seated in a dimly lit room in which luminescence was kept equal throughout the tasks. The eye-tracker was placed 57 cm in front of the participant, just below the computer screen. A chin rest was used to prevent head movement. Additionally, participants were instructed to focus on the screen as much as possible during the tasks, aided by the use of fixation crosses and short duration of blocks. Pupil diameter was recorded at a sampling frequency of 60 Hz.

### Subjective Ratings

After two blocks of each level of difficulty participants indicated on 10-point scales how mentally demanding the task was (cognitive demand), how much they did their best (applied effort), how temporally demanding the task was (time pressure), how well they thought they performed (performance), and how frustrated they were during the task (frustration). These questions were adapted from the NASA Task Load Index (NTLX; Hart and Staveland, 1988). After completing these subjective ratings, the participants were given feedback. We made participants believe that feedback was contingent upon their performance, and that the feedback would indicate whether they performed “better,” “worse,” or “similar to their peers,” but in fact we gave everyone the same feedback (“similar to peers”) after every level of difficulty to prevent feedback-induced differences in motivation between individuals and groups.

Before the *N*-back task, participants were asked to rate their motivation to perform, and their level of fatigue on a 10-point Likert scale. After the *N*-back task, participants again had to indicate their level of fatigue and how much they liked the task.

### Statistical Analyses

#### Performance

In line with signal detection theory (Green and Swets, 1966), as measures of task performance, *perceptual sensitivity* (*d’*) and *response bias* (*c*) were calculated for each level of difficulty of the *N*-back task, on the basis of the frequency of hits, correct rejections, misses, and false alarms across runs (Sorkin, 1999; Karatekin et al., 2009). Higher values of *d’* indicate better ability to discriminate between targets and non-targets/distractors, whereas higher values of *c* indicate greater bias to respond that a target was absent (Karatekin et al., 2009). The ADHD and control group were compared on the values of *d’* and *c* by means of repeated-measures ANOVAs (RM-ANOVAs) using level of difficulty (1-, 2-, 3-, 4-, and 5-back) as within-subject variable, and group (ADHD, control) as between-subject variable.

#### Pupil Dilation

All data were analyzed in R (version 3.3.2; R Core Team, 2016). Pre-processing of the pupil dilation data was done according to a standard procedure (see Supplementary Material). We took the last 500 ms of each preceding 3-s fixation period to baseline the pupil responses during the task blocks by means of subtracting the mean pupil response of the fixation period from the pupil responses in the task block. Since the *N*-back task requires sustained attention, and we did not expect an up- and downregulation of effort on each trial as indexed by pupillary fluctuations, we could examine pupil responses at block level (50-s blocks of 20 trials) rather than at stimulus/trial level.

Due to their non-linear nature (see Supplementary Figures S1, S2), we used generalized additive mixed modeling (GAMM) to examine the time course of pupil size changes (within a 50-s block) by level of difficulty, and group (between subjects). The R packages *mgcv* (Wood, 2006) and *itsadug* (van Rij et al., 2017) were used to implement this analysis. We refer to the Supplementary Material for an introduction to GAMM and a discussion of its advantages as well as the model selection strategy to arrive at a final model.

In our GAMM model, participants were considered as a random effect, and we included a random factor smooth for the time variable to allow for individual variation in the pupil time course for each participant. Furthermore, we included a random intercept and a random slope for the time variable for each trial in the dataset. An autocorrelation parameter was included in our final model. We built a single model in which we could assess all our research questions simultaneously. In the parametric, linear part of our model, we only included a main effect of group to assess whether pupil size was on average smaller or larger in the ADHD group compared to the control group. All other variables of interest (time, difficulty level, and any interactions between variables) were added to the non-parametric part of the model. To model the (potential) non-linear relationship between these predictors and pupil size, we used thin plate regression splines for the smoothing functions (see Supplementary Material for more information).

#### Subjective Ratings

Groups were compared on self-reported experienced cognitive demand, applied effort, performance, time pressure, and frustration during the *N*-back task by means of RM-ANOVAs using level of difficulty and run as within-subject variables, and group as between-subject variable. Groups were also compared on fatigue ratings by means of an RM-ANOVA with time (before, after *N*-back task) as within-subject, and group as between-subject variables. Finally, groups were compared on motivation to perform and liking of the *N*-back task by means of two independent-samples *t*-tests.

### Part II: Effort Discounting

The effort discounting task was also adapted from Westbrook et al. (2013). Participants had to choose repeatedly between performing an easy *N*-back task (1-back, fixed) for a small variable reward and performing a more difficult *N*-back task (2- ,3-, 4-, and 5-back) for a larger fixed reward (2 or 5 euros). This design allowed us to examine how individuals value effortful tasks, and whether individuals with ADHD differ from controls in these cost–benefit analyses. An adjusting-amount procedure was used to reach the point of indifference between the smaller less effortful reward and the larger more effortful reward for each individual [see Westbrook et al. (2013) for details]. Since groups may differ in their sensitivity to reward magnitude, which may influence the steepness of discounting, we included two larger base amounts (for the larger fixed reward): 2 and 5 euros. Participants started with a choice between 1 and 2 euros (2-euro base amount), and 2.50 and 5 euros (5-euro base amount). Participants made five choices for each level of difficulty and each base amount, resulting in a total of 40 choices. The task consisted of 2 runs of 40 choices with a short break in between. Two runs were administered to check potential between-group differences in consistency of choices, since application of an adjusting-amount procedure can easily lead to spurious differences between runs, when someone occasionally responds prematurely or without much consideration.

All choices were potentially real, that is, participants were told that one of their choices was randomly selected by the computer to be performed another four times in an *N-back redo* after completing the effort discounting task, and that they would then receive the corresponding reward. Participants were also told that receipt of the reward during this *N*-back redo was contingent upon exerted effort rather than performance (actual hits), and that effort was monitored during the task using an eye-tracker. Finally, it was emphasized that participants should choose whatever they preferred, and that there were no right or wrong answers. After task instructions, participants were asked to explain the task to the experimenter in their own words to check whether they understood task instructions.

After the effort discounting task, one choice was randomly selected (from the base amount of 5 euros) which, after a short break, was performed two times by the participant. After this *N*-back redo, he received the monetary reward earned (varying between 1^1^ and 5 euros). This amount was added to the financial compensation (20 euros) that participants received for participation and was transferred to their bank account.

After the *N*-back redo, participants completed a short self-developed questionnaire targeting the degree (on 5-point scales) to which their decisions were based on offered amount, difficulty level, desire to do well, desire to challenge themselves, as well as some open questions to learn more about their strategies.

### Statistical Analyses

The adjusting-amount procedure used in the effort discounting task resulted in a subjective value (SV) for each level of difficulty (2-, 3-, 4-, and 5-back task), indicative of the amount at which an individual is indifferent between the effortful and less effortful reward (1-back task). Since two different base reward amounts were used, these SVs were first normalized by expressing them as a proportion of the maximum reward (2 or 5 euros) per effort. A RM-ANOVA was conducted on normalized SV using level of difficulty (2-, 3-, 4-, and 5-back), run (1, 2), and base amount (2 euros, 5 euros) as within-subject variables and group as between-subject variable.

## Results

Two participants (one ADHD, one control) prematurely ended the study during the *N*-back task due to fatigue and/or lack of motivation to continue. Thus, the data from 15 boys with ADHD and 16 controls were used in the analyses (see Table 1 for participant characteristics). It should be noted that on the basis of the DBDRS and CBCL five participants with ADHD did not meet clinical cutoffs for ADHD anymore at the time of testing. Two participants in the ADHD group met clinical cutoffs for ODD, and another two were in the subclinical range on the basis of the DBDRS. None scored in the subclinical or clinical range of CD. None of the controls scored in the (sub)clinical range of ADHD, ODD, or CD. No one was excluded on the basis of DBDRS or CBCL scores.

**Table 1:** Participant characteristics.

	ADHD (*n* = 15)	Controls (*n* = 16)	
Age	15.3 ± 1.5	14.8 ± 1.6	*p* = 0.447
IQ estimate^†^	103.5 ± 7.6	111.4 ± 10.2	*p* = 0.028^∗^
**DBDRS**			
Inattention	15.3 ± 1.9	10.4 ± 0.7	*p* < 0.001^∗∗^
Hyperactivity/impulsivity	14.3 ± 2.4	10.6 ± 1.2	*p* < 0.001^∗∗^
ODD	13.8 ± 2.6	11.0 ± 1.3	*p* = 0.001^∗∗^
CD	11.7 ± 1.6	10.6 ± 1.0	*p* = 0.039^∗^
**CBCL (DSM scales)**		
ADHD problems	67.6 ± 8.7	51.1 ± 2.0	*p* < 0.001^∗∗^
ODD problems	58.2 ± 7.8	51.5 ± 1.9	*p* = 0.005^∗∗^
CD problems	55.3 ± 5.0	51.3 ± 2.3	*p* = 0.009^∗∗^
Affective problems	58.3 ± 7.3	50.6 ± 1.3	*p* = 0.001^∗∗^
Anxiety problems	58.7 ± 7.0	51.6 ± 2.2	*p* = 0.002^∗∗^
Somatic problems	53.3 ± 4.2	51.5 ± 2.7	*p* = 0.163
Reward sensitivity (BIS/BAS)	17.7 ± 1.7	16.9 ± 2.1	*p* = 0.261
Need for cognition (NCS)	54.2 ± 8.0	56.3 ± 7.5	*p* = 0.467


*DBDRS, Disruptive Behavior Disorders Rating Scale (parent version); standardized scores: ≤14 normal range, 15 subclinical range, ≥16 clinical range for inattention and hyperactivity/impulsivity, ≤15 normal range, 16 subclinical range, and ≥17 clinical range for Oppositional Defiant Disorder (ODD) and Conduct Disorder (CD); CBCL, Child Behavior Checklist (parent version), *T*-scores for DSM scales: 50–64 normal range, 65–69 borderline clinical range, and 70–100 clinical range; BIS/BAS, Behavioral Inhibition/Approach System scales; NCS, revised Need for Cognition Scale; ^†^missing data from three participants (one ADHD, two controls); ^∗^*p* < 0.05, *^∗∗^p* < 0.01.*

### Part I: Performance *N*-Back Task With Varying Degrees of Task Difficulty

**FIGURE 1:**
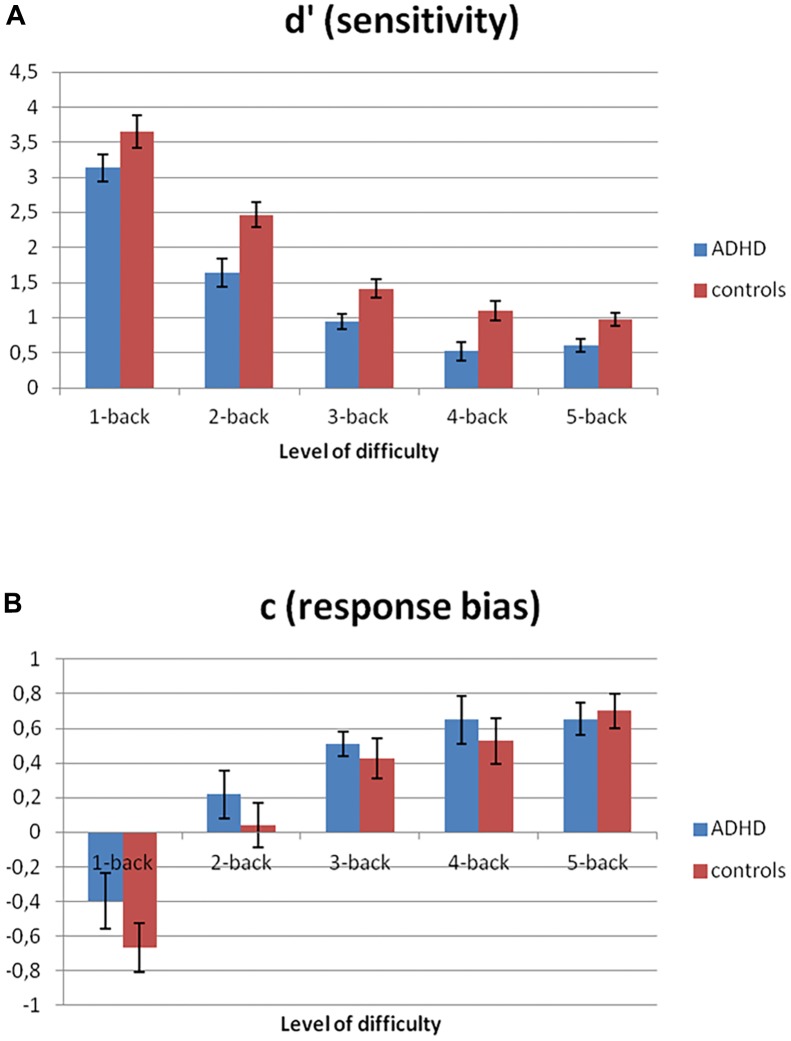
Performance on *N*-back task. Participants with ADHD **(A)** are overall worse at distinguishing target from non-target letters (*d’*) and **(B)** have similar response bias (c) toward choosing “non-target letter” with increasing task difficulty.

Overall performance deteriorated as task difficulty increased [*d’* sensitivity; *F*(4,116) = 147.6, *p* < 0.001, $\eta_{p}^{2}$ = 0.84), and individuals with ADHD performed more poorly than controls [*F*(1,29) = 12.8, *p* = 0.001, $\eta_{p}^{2}$ = 0.31]. However, there was no interaction between group and level of difficulty on *d’* [*F*(4,116) = 0.92, *p* = 0.43, $\eta_{p}^{2}$ = 0.03] – that is the effect of increasing task difficulty was not larger for ADHD than control participants (Figure 1A). Taking IQ into account as a covariate did not change the results [main effect of group: *F*(1,25) = 8.62, *p* = 0.007, $\eta_{p}^{2}$ = 0.26]. There was also a significant overall main effect of task difficulty on response bias *c* [*F*(4,116) = 45.1, *p* < 0.001, $\eta_{p}^{2}$ = 0.61] but no effect of group [*F*(1,29) = 1.0, *p* = 0.32, $\eta_{p}^{2}$ = 0.03] or interaction with group [*F*(4,116) = 0.64, *p* = 0.61, $\eta_{p}^{2}$ = 0.02) (Figure 1B).

### Pupil Responses

**FIGURE 2:**
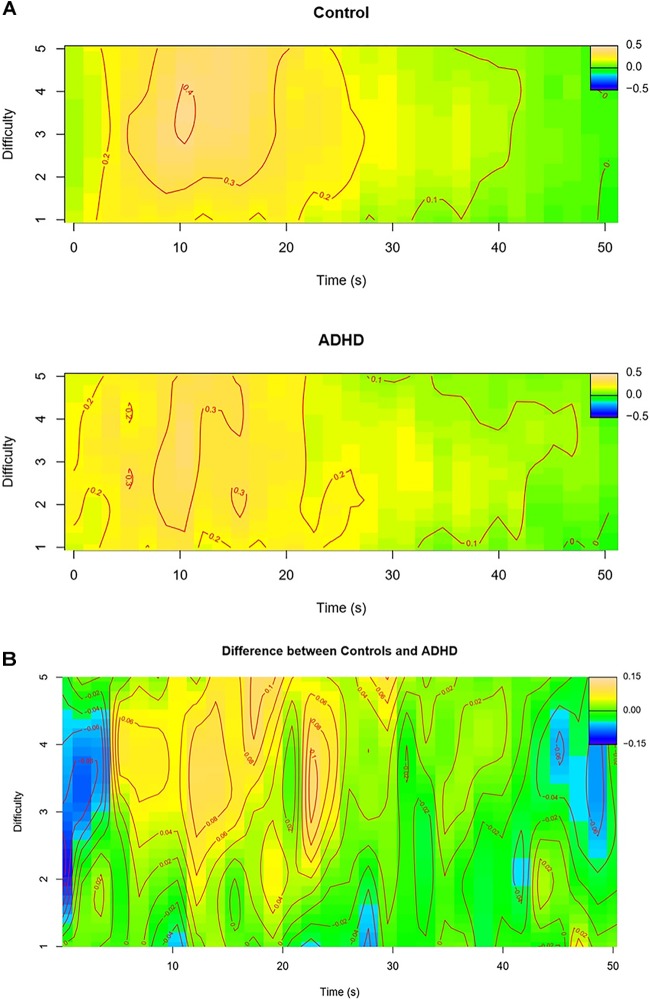
Difference surface plots for pupil responses during *N*-back tasks. **(A)** Surface plots for the control and ADHD group separately, showing that peak pupil dilation occurs within approximately 10 s after task onset (yellow-red areas), and appears highest for the control group for intermediate levels of difficulty (3-, 4-back), and is more diffuse for the ADHD group across levels of difficulty. **(B)** Surface plot showing the difference in pupil size between the control and ADHD group, which is most apparent on intermediate and higher levels of difficulty (3-, 4-back) between 5 and 25 s after task onset (yellow–red areas; controls > ADHD) (blue areas: ADHD > controls).

Table 2 summarizes the parameter estimates of the GAMM model that was fitted to the data. The random factor smooth for time was significantly nonlinear, indicating individual differences in time course for different participants. Furthermore, the random intercepts and slopes indicated trial-to-trial variability in the average pupil size as well as in the steepness of the nonlinearity. Looking at the main effects of interest there were no main effects of group (ADHD/control) or task difficulty and no interaction between group and difficulty. There was, however, a significant non-linear effect of time on pupil size and a significant three-way interaction between group, task difficulty, and time. Figure 2A visualizes the non-linear interaction between time and difficulty separately for each group and Figure 2B depicts the differences between groups. This shows that the largest differences in pupil dilation occur during the more difficult *N*-back levels. Early on in a trial-block, the pupil size differences are slightly negative, indicating that pupil size is larger for participants with ADHD than controls. This difference, however, quickly reverses sign: after about 5 s in a block, pupil size is larger for controls than for those with ADHD, which is quite sustained until about 25 s have passed. After 25 s, there are no pronounced differences anymore between groups.

**Table 2:** Summary of the parametric and non-parametric part of the GAMM model.

Parametric coefficients	Estimate	Std. error	*t*-value	*p*-value
Group	-0.0083	0.0407	-0.2048	0.8377

**Non-parametric coefficients**	**EDF**	**Ref. df**	***F*-value**	***p*-value**

Time (s)	171.3972	189.8314	12.1397	<0.0001^∗^
Difficulty (s)	2.1324	2.1816	2.0582	0.0991
Time × group (s)	136.0455	166.9725	4.1729	<0.0001^∗^
Difficulty × group (s)	1.0116	1.0125	0.3394	0.5721
Difficulty × time (ti)	154.7116	176.8799	5.2048	<0.0001^∗^
Difficulty × time × group (ti)	147.0235	175.3145	3.5023	<0.0001^∗^
Random smooth for time (s)	188.4874	276.0000	399.5384	<0.0001^∗^
Random intercept for time (s)	483.3168	608.0000	48.3211	0.0098^∗^
Random slope for time (s)	374.6543	608.0000	64.2624	0.0091^∗^


*EDF, estimated effective degrees of freedom; Ref. df, alternative degrees of freedom; s, smooth function; ti, tensor product interaction; ^∗^*p* < 0.001.*

### Subjective Ratings

**FIGURE 3:**
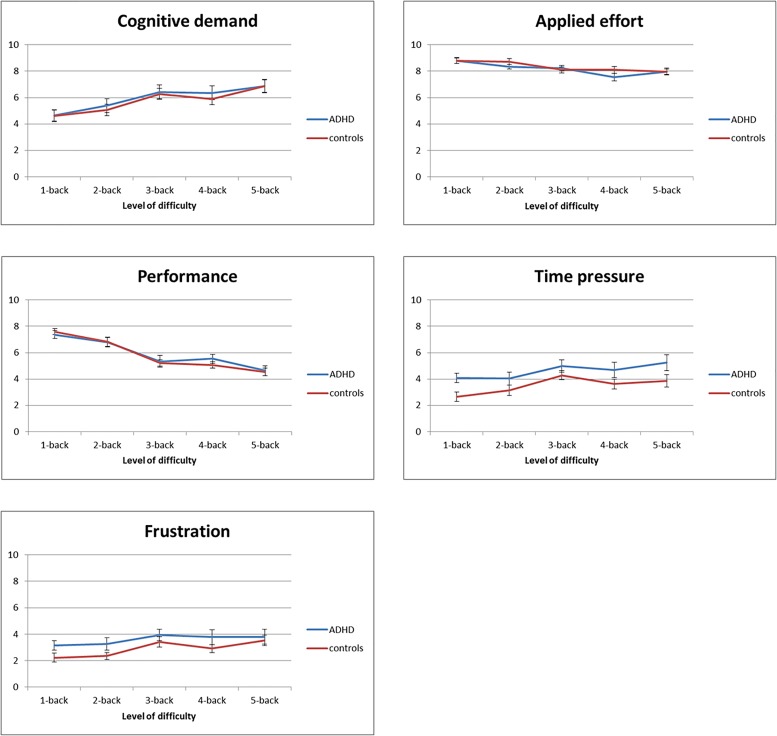
Subjective ratings associated with each level of difficulty of the *N*-back task averaged across both runs. Cognitive demand, time pressure, and frustration increased with difficulty, while applied effort and performance decreased. No significant group differences were found.

With increasing level of *N*-back difficulty, participants experienced, as expected, an increase in cognitive demand [*F*(4,112) = 26.5, *p* < 0.001, $\eta_{p}^{2}$ = 0.49]^2^, time pressure [*F*(4,116) = 8.8, *p* < 0.001, $\eta_{p}^{2}$ = 0.23], and frustration [*F*(4,116) = 7.6, *p* < 0.001, $\eta_{p}^{2}$ = 0.21], and a decrease in performance [*F*(4,116) = 66.8, *p* < 0.001, $\eta_{p}^{2}$ = 0.70]. Against expectations, perceived applied effort decreased with increasing task difficulty [*F*(4,116) = 14.6, *p* < 0.001, $\eta_{p}^{2}$ = 0.33] (Figure 3). In addition, there were effects of run: in the second run, cognitive demand was lower, participants perceived less time pressure and were less frustrated, while performance was rated better than in the first run. Applied effort did not significantly decrease from the first to the second run (*p* = 0.051). Importantly, there were no significant group differences in subjective ratings (all *p*s > 0.052; lowest *p*-value for time pressure), except for an interaction between group and run on frustration [*F*(1,29) = 5.2, *p* = 0.030, $\eta_{p}^{2}$ = 0.15]: the difference (i.e., decrease) in level of frustration between the first and second run was larger for the ADHD group than for the control group.

Fatigue ratings were higher after the *N*-back task (*M* = 4.6, *SD* = 1.6) than before (*M* = 3.3, *SD* = 1.6) [*F*(1,29) = 16.6, *p* < 0.001, $\eta_{p}^{2}$ = 0.36]. Groups did not differ in fatigue ratings, neither did they differ in self-reported motivation preceding the *N*-back task and liking of the task.

### Part II: Effort Discounting

**FIGURE 4:**
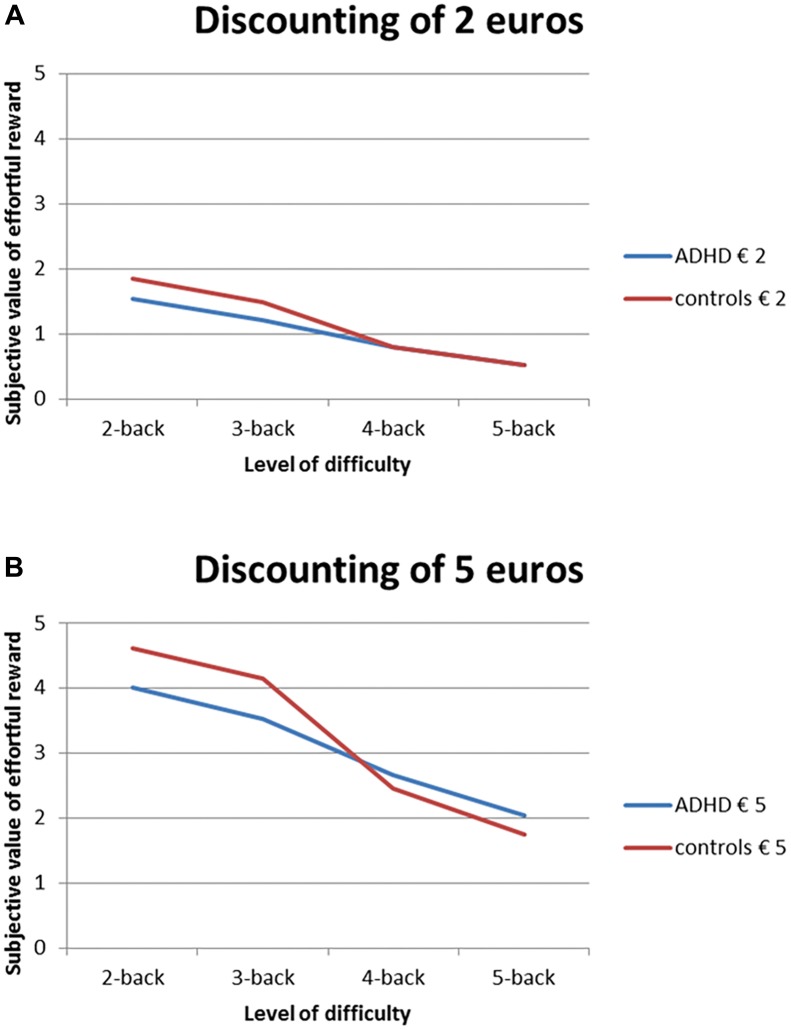
Non-normalized SVs of rewards (i.e., indifference points) associated with the effortful option (2-, 3-, 4-, and 5-back against 1-back) averaged for both runs for base amounts **(A)** 2 euros and **(B)** 5 euros. Both groups display effort discounting, and groups did not differ significantly from each other.

Four participants (two ADHD, two controls) responded prematurely in at least one of the trials, i.e., before the choice options were presented^3^, leading to an unreliable SV. The data of these participants were therefore discarded. For the remaining participants, we found a main effect of difficulty on SV [*F*(3,75) = 76.6, *p* < 0.001, $\eta_{p}^{2}$ = 0.75), showing that rewards associated with more effort were discounted, as expected (Figure 4). We also found an effect of amount [*F*(1,25) = 19.7, *p* < 0.001, $\eta_{p}^{2}$ = 0.44] and an interaction between difficulty and amount [*F*(3,75) = 2.9, *p* = 0.042, $\eta_{p}^{2}$ = 0.10]: the larger base amount (5 euros) was discounted less, and less steeply than the smaller base amount (2 euros). In other words, the larger base reward does not lose its value as quickly as the smaller base reward, i.e., participants are more likely to apply effort to obtain that reward. Importantly, there was no main effect of group [*F*(1,25) = 0.84, *p* = 0.369, $\eta_{p}^{2}$ = 0.03], neither were there any interactions with group (all *p*s > 0.20), suggesting that effortful rewards are discounted in a similar way by boys with ADHD and controls. Also, no effects of run were found (all *p*s > 0.26), suggesting that choices were not random.

No significant group differences (all *p*s > 0.12) were found on our self-developed questionnaire targeting the degree to which decisions were based on offer amount, difficulty level, desire to do well, and desire to challenge oneself.

## Discussion

In this pilot study we examined ADHD-related alterations in two effort-related functions: effort application and effort-related decision-making, using a multi-modal approach. As expected, with increasing level of *N*-back difficulty, performance deteriorated, and boys with ADHD performed significantly worse than controls. Against expectations, however, this worse performance was stable across difficulty levels. Groups also did not differ in their perception of mental demand and effort application, and appeared to have similar cost–benefit analyses outcomes regarding effortful rewards. Differential effects of increasing effort were, however, seen on pupil dilation data where results may indicate different effort allocation in the ADHD group compared to the control group.

First of all, our finding that increasing task load did not lead to increasingly worse performance in boys with ADHD compared to controls suggests that they are unlikely to suffer from an effort allocation deficit. We predicted that group differences would become more apparent with increasing task load requiring more effort, but this was not the case. This finding also suggests that they are unlikely to have a shortage of working memory capacity. Previous studies showing similar results suggested that task demands might not have been high enough to exhaust children’s working memory capacity (Gomarus et al., 2009; Van De Voorde et al., 2011), but in the current study the highest levels of *N*-back difficulty must have been demanding enough to reveal a shortage of capacity. Since the ADHD group performed worse than controls on all levels of *N*-back difficulty, it is more likely that they suffer from a basic information processing deficit (see also Metin et al., 2013; Salum et al., 2014).

The pupil data showed a group difference, but only in relation to level of difficulty and time-on-task. The control group showed a pattern suggesting that more effort is allocated with increasing task load up to a point where this is still feasible (and appear to show disengagement at the highest level of difficulty). The ADHD group, on the other hand, showed a more diffuse pattern, i.e., they appeared to allocate more similar amounts of effort to all difficulty levels (Figure 2A). The group effects are therefore most apparent in the intermediate to higher levels of difficulty: in these blocks, while participants with ADHD showed larger pupil sizes than controls in the first seconds of the task (blue areas in Figure 2B), controls showed larger pupil sizes between 5 and 25 s (yellow areas), suggesting that they increase their effort more than those with ADHD. This could thus imply an effort allocation deficit in ADHD participants. They may not be able to allocate their effort in line with the demands of the task. Alternatively, since performance did not worsen more with increasing task load in ADHD, we could argue that they were better at keeping their performance at a certain level with less effort allocation than controls. On the basis of these subtle group differences in pupil patterns – in combination with a lack of a similar group difference in behavior – it is difficult to draw strong conclusions regarding an effort allocation difference between ADHD and control participants.

In order to provide a more conclusive answer to the question whether the group difference in pupil response patterns reflects an effort allocation deficit in ADHD, it is necessary to directly link these patterns to performance. Unfortunately, the current (block) design does not allow us to directly test this association, because performance on the *N*-back tasks (defined as *d’*) was highly correlated with level of difficulty as can been seen from Figure 1A. This prevented us from reliably distinguishing between the variance explained by performance and by difficulty when both factors would be included in the GAMM model. Extracting a single measure from the pupil dilation data to directly correlate with *d*’ is also difficult. On the basis of our behavioral results, however, one would expect a similar response pattern for the ADHD and control group across all levels of difficulty, albeit a generally decreased pupil size in ADHD, which is not what we found. It is likely though that the method used for the pupil analyses was more sensitive to pick up subtle group differences than the method used for our performance analysis. In sum, despite group differences in pupil responses depending on task difficulty and time-on-task, *and* a main group difference in performance, on the basis of these results, we are not able to draw conclusions on whether the two are directly related. Future studies are needed to directly test this association.

In addition to the abovementioned objective measures of effort, we also examined participants’ subjective experiences of effort. Subjective ratings of mental demand were similar for both groups, in contrast to a study by Hsu et al. (2017) who found that undergraduates at risk for ADHD reported higher mental effort than those not at risk. In this latter study, both groups performed equally on the task, while in our study the ADHD group performed worse. The discrepancy may lie in the fact that in the Hsu et al. (2017) study participants were highly educated, high-functioning individuals who were at risk for ADHD, while our study included adolescent boys with an ADHD diagnosis. In order to perform at the same level of their peers, high-functioning at-risk individuals may indeed have put in more effort and found the tasks more mentally demanding than others, but were capable of upregulating or allocating their effort effectively. There is, however, an increasing body of evidence suggesting that individuals diagnosed with ADHD overestimate – and thus lack insight into – their performance (for a review, see Jiang and Johnston, 2014). Indeed, there was no group difference in *perceived* performance in the current sample, while there was in *actual* performance. This positive illusory bias may extend to estimates of mental demands and effort expended on a task, which may explain our lack of group differences in these subjective ratings.

Across groups, the mental demand ratings showed the expected increase with increasing task load. Applied effort ratings, however, *decreased* with higher task load. This unexpected finding may be explained by decreased motivation to perform on the more difficult tasks, and may resonate with participants’ perceived performance on the task. A ceiling effect may also have contributed to this finding, since participants started with the easiest task (1-back) and reported high levels of applied effort to begin with, leaving little room for improvement. Indeed, while the effect of task load on applied effort was statistically significant, variation in this self-reported applied effort was low. This is in line with the findings by Mulert et al. (2007) who found self-reported applied effort (“volitional mental effort”) to be rather constant across variations in task difficulty, while mental demand (“mental effort demands”) increased with task difficulty.

In line with these subjective experiences, we found no group difference in the subjective cost of effort. In other words, participants with ADHD were just as willing to perform an effortful task for money than controls. This argues against the “effort aversion” hypothesis, and is in line with two recent studies on physical effort in children with ADHD (Winter et al., 2016; Mies et al., 2018). In the study by Winter et al. (2016), children with ADHD made an equal amount of high-effort choices as controls. They were, however, less capable of exerting the physical effort associated with their choices. It is also possible that the use of monetary incentives in effort discounting tasks has a stronger impact on motivation in individuals with ADHD than controls, leading to similar cost–benefit *outcomes* for both groups, despite the cost–benefit analysis itself being different. Our null-finding with respect to group differences in the degree to which decisions were made based on offered amount and difficulty level, however, do not point in this direction. Also no group difference was found on the NCS (Table 1), suggesting that the boys with ADHD in our sample were just as interested in activities that require thinking (i.e., mental effort) than their non-affected peers. It is thus more likely that they were unaware of their worse performance on the *N*-back tasks (and perhaps diminished ability to allocate their effort), and, probably as a consequence, made similar choices as their non-affected peers (see also Winter et al., 2016). This suggests that the adolescent boys with ADHD in our sample did not have a motivational problem with conducting effortful tasks. As concluded by Winter et al. (2016), children with ADHD may not have different cost–benefit analyses related to effort, but they do seem to have difficulties in performing, or allocating effort to implement their preferences.

Together, the results of this pilot study suggest that adolescent boys with ADHD may not have a motivational problem with applying effort, or in other words, are not necessarily effort averse, but on the basis of the pupil findings, they do seem to differ from controls in allocating effort during demanding tasks. In terms of state regulation accounts of ADHD, these results may imply that individuals with ADHD do not have difficulties with effort application *per se*, i.e., *cognitive*/*computational* effort to perform a (demanding) task (Mulder, 1986), since increasing working memory load did not lead to larger group differences. Previous studies, however, have focused on the state regulation deficit hypothesis using event rate manipulations, and provide evidence for effort allocation difficulties in ADHD when additional effort is needed to regulate activation and arousal. The latter is known as *compensatory* effort to protect performance under demanding task conditions (Fairclough and Mulder, 2011). Our pupil findings may be in line with such compensatory effort allocation difficulties in ADHD, as our ADHD group appears to show less up- and downregulation of effort in line with mental demand within (more difficult) trial blocks than our control group. The distinction between these two mechanisms – or levels – of action of mental effort is important here (see also Sanders, 1983): effort needed to upregulate activation and arousal (at a trial-to-trial basis) vs. effort directly linked to the central stages of information processing, on which working memory load acts. Future studies could address this distinction by examining both compensatory effort (as focused on in event rate studies) and computational effort (as in the current working memory task study with different levels of difficulty) in the same individuals.

This is, to our knowledge, the first study that examined the role of mental effort in ADHD taking a multi-modal approach by looking at performance, self-report ratings, physiological responses, and the subjective cost of effort as a function of reward. The main limitation of this study is the small sample size, which may have resulted in less power to find group differences, especially in the effort discounting task, since we had to exclude a few extra participants for these analyses. We should thus be cautious when interpreting and extrapolating these results to children/adolescents with ADHD in general. Another limitation is the lack of motivation ratings during the *N*-back tasks. It would have been informative to know whether our feedback manipulation to keep motivation equal for everyone worked, and whether there were group differences in motivation vis-à-vis task load. In addition, since ADHD is a heterogeneous disorder (e.g., Kofler et al., 2017), and it is unlikely that all individuals with ADHD will suffer from an effort allocation deficit, future studies with larger sample sizes are needed to replicate these results, and examine whether these findings can be linked to specific subtypes of ADHD, and/or to comorbid conditions such as ODD and CD. Not having been able to appropriately link the behavioral findings directly to the pupil findings is another limitation of this study. A different task set-up suited to examine effort on a trial-to-trial basis would be effective in doing so. Finally, we cannot rule out effects of long-term medication use and short-term withdrawal of this medication on our findings. Methylphenidate acts by blocking the dopamine (and norepinephrine) transporter, and dopamine is known to play a role in motivation and effort (e.g., Salamone et al., 2016). It is thus needed to take medication status into account in future studies on effort in ADHD, preferably testing individuals both on and off medication.

To conclude, increasing the requirement for additional effort on a working memory task affected performance, preferences, and perceptions of task demands, and effort application of individuals with ADHD and controls in similar ways. Differential effects of increasing effort were, however, seen in pupil dilation data where results suggested different effort allocation in the ADHD group. Future studies are needed to see if these results can be replicated in larger samples, and in order to explore the source of the discrepancy between physiological, behavioral, and subjective measures of effort.

## Ethics Statement

All participants and their parents gave written informed consent prior to participation. The study was conducted in accordance with the Declaration of Helsinki. The protocol was approved by the local medical ethics committee of UZ Leuven, Belgium (S57756).

## Author Contributions

GM, ES-B, MD, and SvdO were the main contributors to the design of the study. JW, AS, and JL provided valuable input on the design of the study. GM collected the data with the help from students, analyzed the behavioral data, and took the lead in writing the manuscript. PM programmed the experimental tasks, conducted the pupil analyses, and wrote the corresponding data analysis and results section. ES-B was a major contributor in writing the manuscript and interpretation of the results. MD, JW, SvdO, and AS gave valuable input on the analyses, interpretation of the results, and writing of the manuscript. All authors critically read and approved the final manuscript.

## Conflict of Interest Statement

SvdO was a paid speaker (Shire, MEDICE) and co-developer of a cognitive training game “Braingame Brian” and two cognitive-behavioral treatments “Plan my Life” and “Solution Focused Treatment” (non-financial interest). ES-B received speaker fees, research funding, and conference support from and has served as consultant to Shire Pharma and received speaker fees from Janssen-Cilag. He served as consultant to Neurotech Solutions, Aarhus University, Copenhagen University and Berhanderling, Skolerne, Copenhagen, and KU Leuven. He has received royalties from Oxford University Press and Jessica Kingsley. MD was a paid member of advisory boards for Shire and Neurotech Solutions, a paid speaker at conferences supported by Shire, Novartis, Medice, and a consultant for Neurotech Solutions. The remaining authors declare that the research was conducted in the absence of any commercial or financial relationships that could be construed as a potential conflict of interest.

## Footnotes

1 0.16 euros was the lowest amount selected by the computer, which occurred twice. These participants received 1 euro instead.

2 Cognitive demand ratings from one boy with ADHD were unreliable and therefore discarded.

3 Due to a programming flaw, button presses were allowed to be recorded *before* stimulus presentation. This was adjusted upon discovery to prevent this issue for subsequent participants.
