# Supplementary material for: A Pilot Study of Behavioral, Physiological, and Subjective Responses to Varying Mental Effort Requirements in Attention-Deficit/Hyperactivity Disorder
Source: Front Psychol. 2019 Jan 11;9:2769. doi: 10.3389/fpsyg.2018.02769 (PMC6336710; doi:10.3389/fpsyg.2018.02769)
Supplement: Supplementary file 1 [file Data_Sheet_1.docx]

**Supplement**

**Pre-processing of pupil data**

The following steps were taken to pre-process pupil responses. First, timestamps of each N-back block were binned into bins of 1/60s because not all data were sampled in an evenly spaced manner. Signal loss or eye blinks were detected through visual inspection and a cut-off of 1 (arbitrary units) was used to discard these data points. If a single 50 second block contained more than 30% missing data, we removed that block from the analysis (1 3-back block from 1 ADHD participant). Because the statistical methods we used to analyse the data can cope well with missing data points, we chose not to interpolate missing data, nor smooth any of the pupil time courses.

**GAMM**

GAMM allows to model the data at the trial level, without having to resort to averaging different pupil traces across blocks or sessions to yield event-response functions similar to those used in event-related potential analysis. GAMM can be seen as an extension to generalized linear mixed modelling (GLMM) through the addition of a non-parametric element to the parametric part of the model. This non-parametric element allows nonlinear smoothing functions to be fit to models so-called “wiggly” lines for a single numerical variable, or “wiggly” surfaces for two or more numerical variables (e.g., interactions). To ensure neither under- nor over-smoothing, a penalty value is added to the fitting routine – the value of which is chosen by generalized cross-validation. As a single block of trials contained only 20 events, we expected that over-smoothing might be an issue in our dataset. Therefore, we took some care to check the number of basis dimensions for the smoothing functions with a procedure implemented in the *mgcv* package. That is, we first fitted models without changing the number of basis dimensions, and verified whether the estimated effective degrees of freedom (EDF) for all terms were close to the default maximum. If so, we increased the number of basis dimensions until the estimated EDF no longer meaningfully changed.

As in traditional GLMM models, GAMM models also contain a fixed and a random effects part. Participants were considered as a random effect, and we included a random factor smooth for the time variable to allow for individual variation in the pupil time course for each participant. Furthermore, we included a random intercept and a random slope for the time variable for each trial in the dataset. We took this approach to reduce residual autocorrelation as much as possible. In addition, we included an autocorrelation parameter in our final model. The estimate of this parameter was based on the autocorrelation function we observed in the error residuals after fitting our final model without this autocorrelation parameter (ρ = .994). Because group differences were of most interest to us, we used a specific approach based on ordered factors to test these (see 60, 61). The reasoning behind this approach is that one models so-called reference curves and/or surfaces for one group of participants (in our case, the control participants). A difference curve and/or surface is then estimated for the other group of participants by modelling an interaction with the variable of interest. By doing so, one can directly interpret the significance of the terms associated with the interaction. If the difference curve/surface is statistically significantly different from zero, one can conclude that the groups differ on that variable. As our experimental design was relatively straightforward, we built a single model in which we could assess all our research questions simultaneously. In the parametric part of our model, we only included a main effect of group to assess whether pupil size was on average smaller or larger in the ADHD group compared to the control group. All other variables of interest were added to the non-parametric part of the model. To model the (potential) non-linear relationship between these predictors and pupil size, we used thin plate regression splines for the smoothing functions. A smooth term for time was included to capture the non-linear pattern of changes in pupil size over time. As we already indicated above, a random factor smooth was also included for the time variable (for the participant random effect) to capture systematic variation in these time courses for different participants. In addition, random intercepts and slopes for the time variable were also included for each separate trial. Because we did not want to assume that the effect of difficulty on pupil size would be linear or non-linear, we also included a smooth term for difficulty level of the N-back task. Interactions with the group variable were included to assess whether these main effects of time and difficulty were different for both groups. In addition, we included a tensor product interaction between time and difficulty to assess whether the effect of difficulty on pupil size varied over time. For this interaction surface, we again tested whether these were different between groups by including an interaction with the group variable.

**
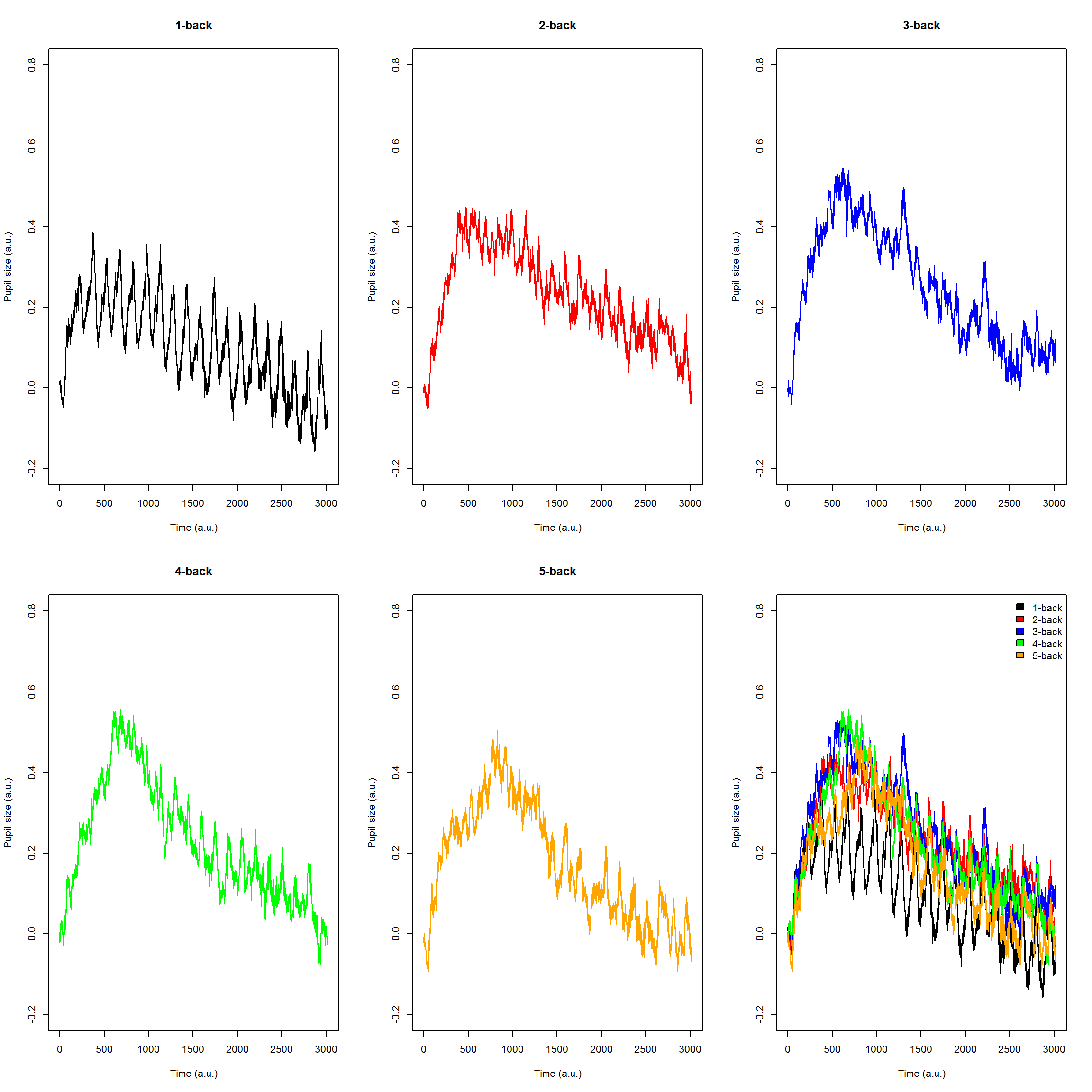
**

**Figure S1** Baseline-corrected average pupil traces for each level of difficulty (individually and plotted together) for the control group

**
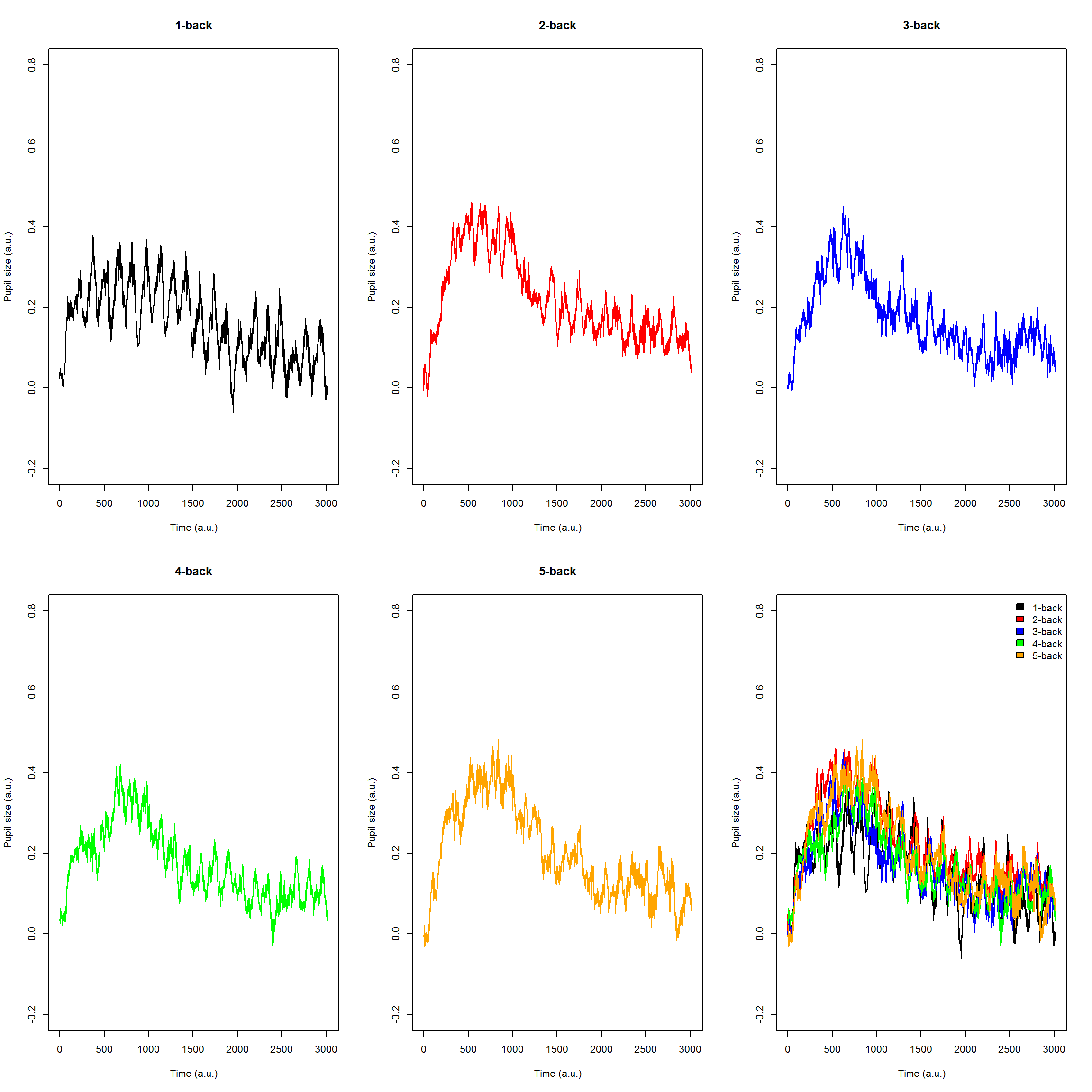
**

**Figure S2** Baseline-corrected average pupil traces for each level of difficulty (individually and plotted together) for the ADHD group
